# Supplementary material for: Rampant Interkingdom Horizontal Gene Transfer in Pezizomycotina? An Updated Inspection of Anomalous Phylogenies
Source: Int J Mol Sci. 2025 Feb 20;26(5):1795. doi: 10.3390/ijms26051795 (PMC11898892; doi:10.3390/ijms26051795)
Supplement: Supplementary file 1 [file ijms-26-01795-s001.zip › Supplementary_Figures.pdf]

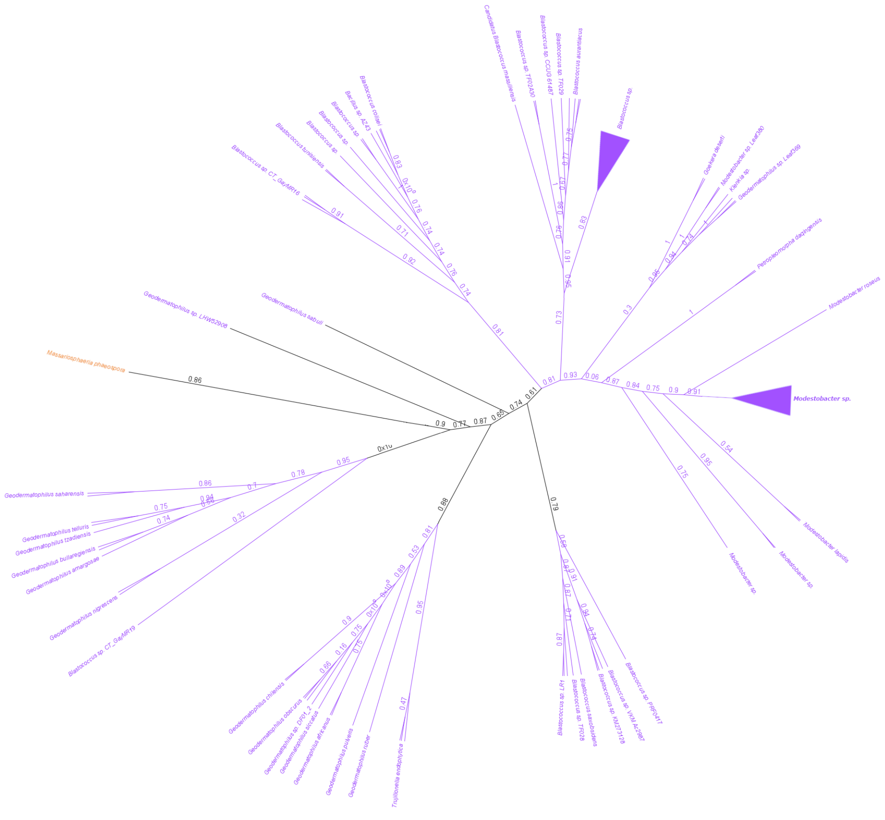

Figure S1. Phylogenetic tree illustrating the HGT 1 event. Fungal species are depicted in black, while non-fungal species are shown in purple, and the original species where the event was observed is indicated in dark yellow. Shimodaira-Hasegawa values are displayed next to the nodes, reflecting the support for the respective clades. For simplicity, some branches have been collapsed and are represented by triangles.

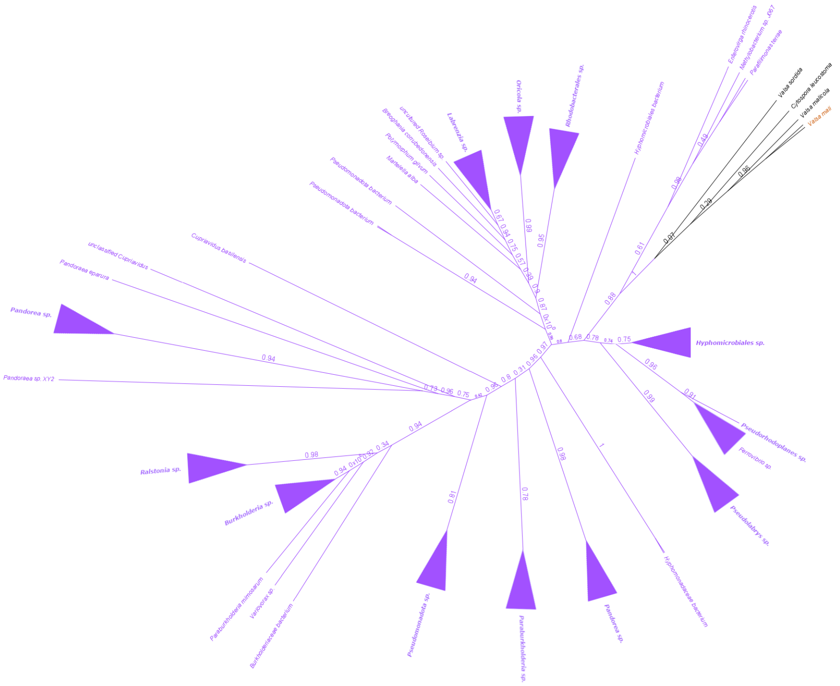

Figure S2. Phylogenetic tree illustrating the HGT 2 event. Fungal species are depicted in black, while non-fungal species are shown in purple, and the original species where the event was observed is indicated in dark yellow. Shimodaira-Hasegawa values are displayed next to the nodes, reflecting the support for the respective clades. For simplicity, some branches have been collapsed and are represented by triangles.

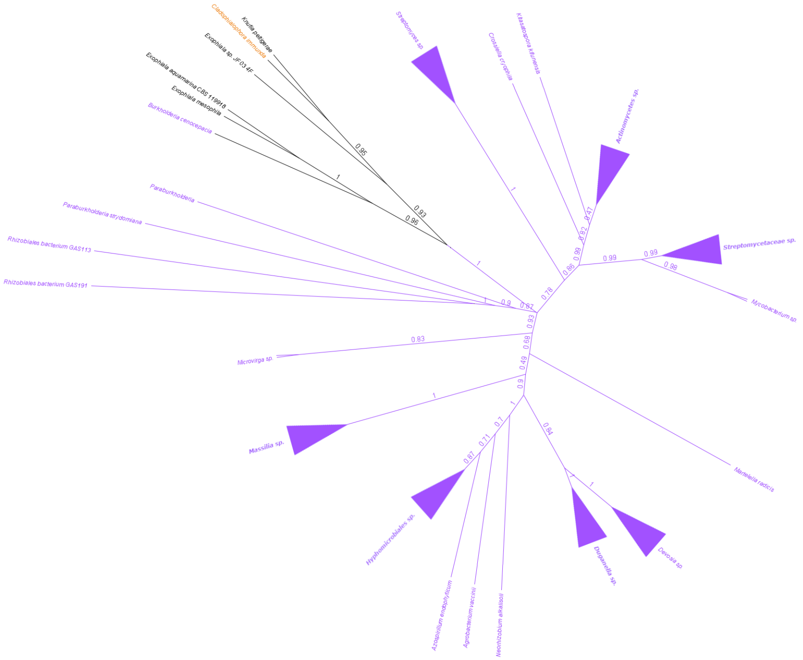

Figure S3. Phylogenetic tree illustrating the HGT 3 event. Fungal species are represented in black, while non-fungal species are shown in purple, and the original species in which the event was observed is indicated in dark yellow. Shimodaira-Hasegawa values are displayed next to the nodes, reflecting the support for the respective clades. For simplicity, some branches have been collapsed and are represented by triangles.

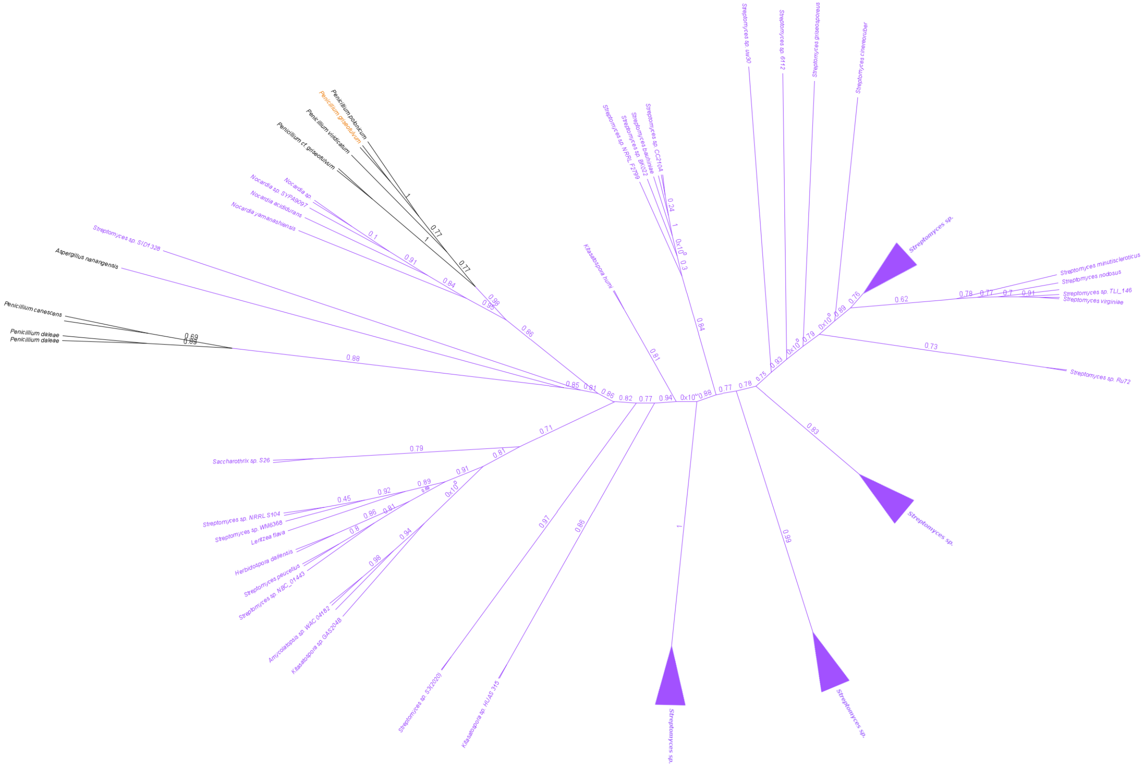

Figure S4. Phylogenetic tree illustrating the HGT 4 event. Fungal species are depicted in black, while non-fungal species are shown in purple, and the original species where the event was observed is indicated in dark yellow. Shimodaira-Hasegawa values are displayed next to the nodes, reflecting the support for the respective clades. For simplicity, some branches have been collapsed and are represented by triangles.

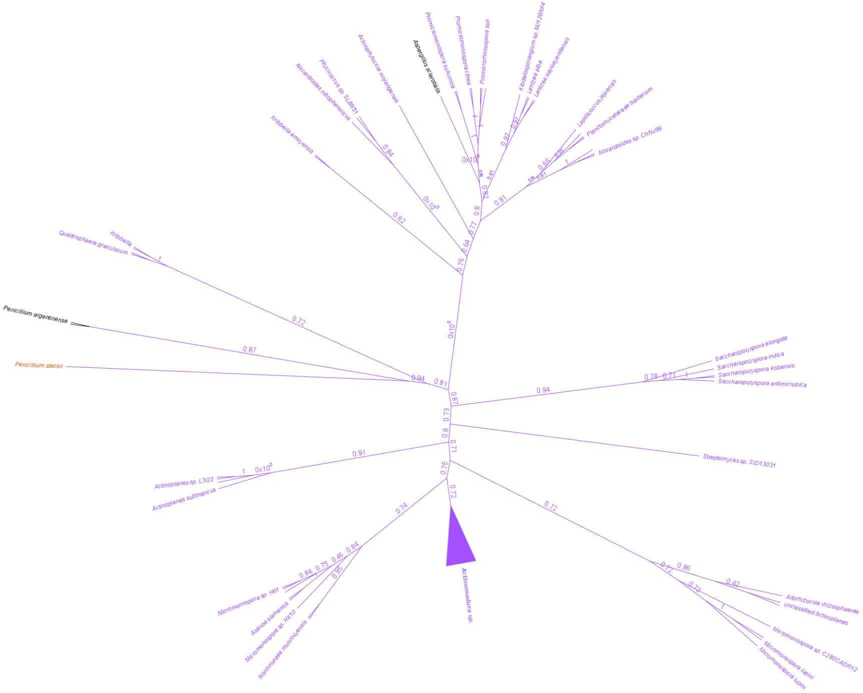

Figure S5. Phylogenetic tree illustrating the HGT 5 event. Fungal species are depicted in black, while non-fungal species are shown in purple, and the original species where the event was observed is indicated in dark yellow. Shimodaira-Hasegawa values are displayed next to the nodes, reflecting the support for the respective clades. For simplicity, some branches have been collapsed and are represented by triangles.

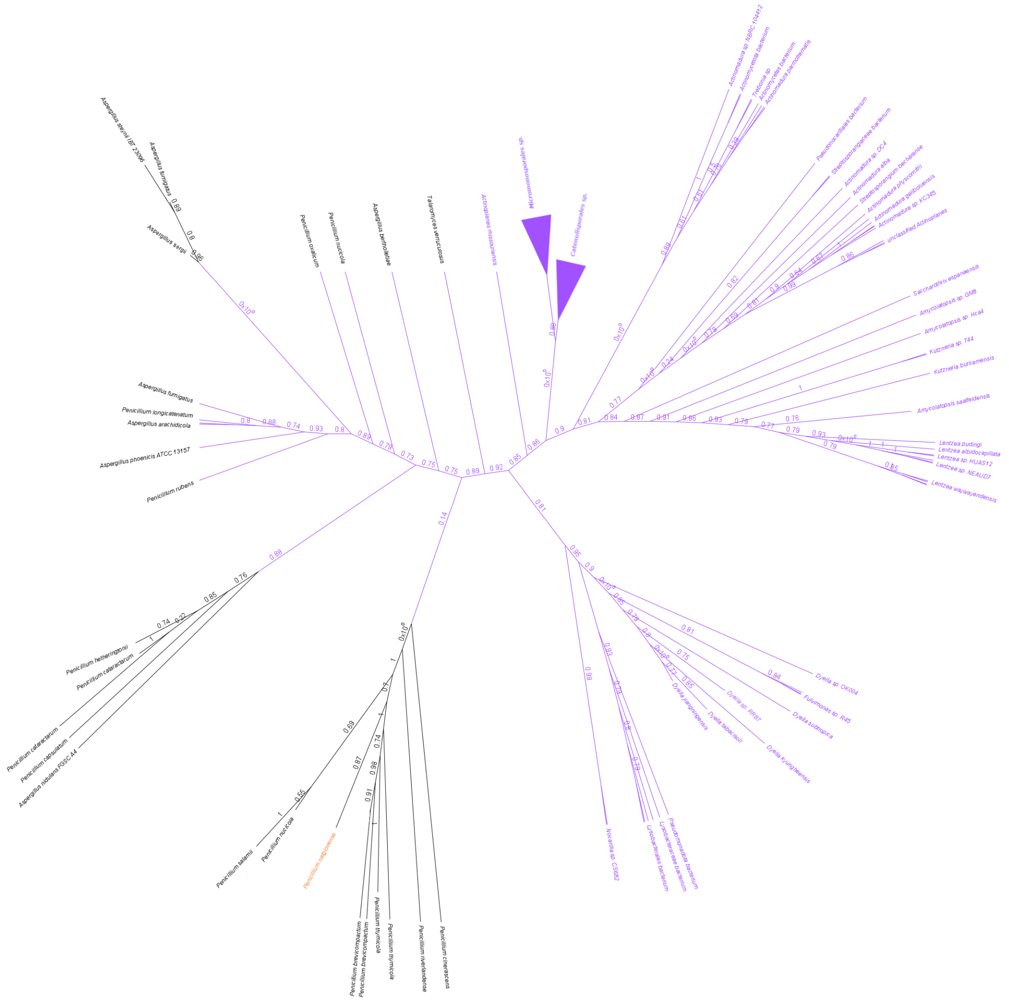

Figure S6. Phylogenetic tree illustrating the HGT 6 event. Fungal species are depicted in black, while non-fungal species are shown in purple, and the original species where the event was observed is indicated in dark yellow. Shimodaira-Hasegawa values are displayed next to the nodes, reflecting the support for the respective clades. For simplicity, some branches have been collapsed and are represented by triangles.

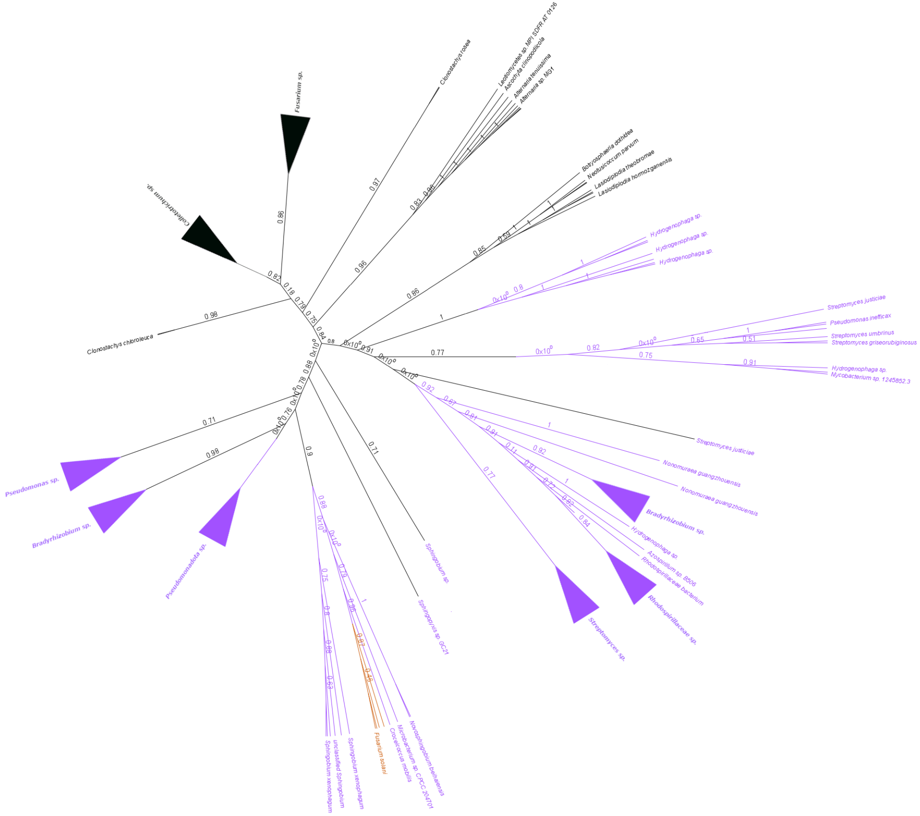

Figure S7. Phylogenetic tree illustrating the HGT 7 event. Fungal species are depicted in black, while non-fungal species are shown in purple, and the original species where the event was observed is indicated in dark yellow. Shimodaira-Hasegawa values are displayed next to the nodes, reflecting the support for the respective clades. For simplicity, some branches have been collapsed and are represented by triangles.

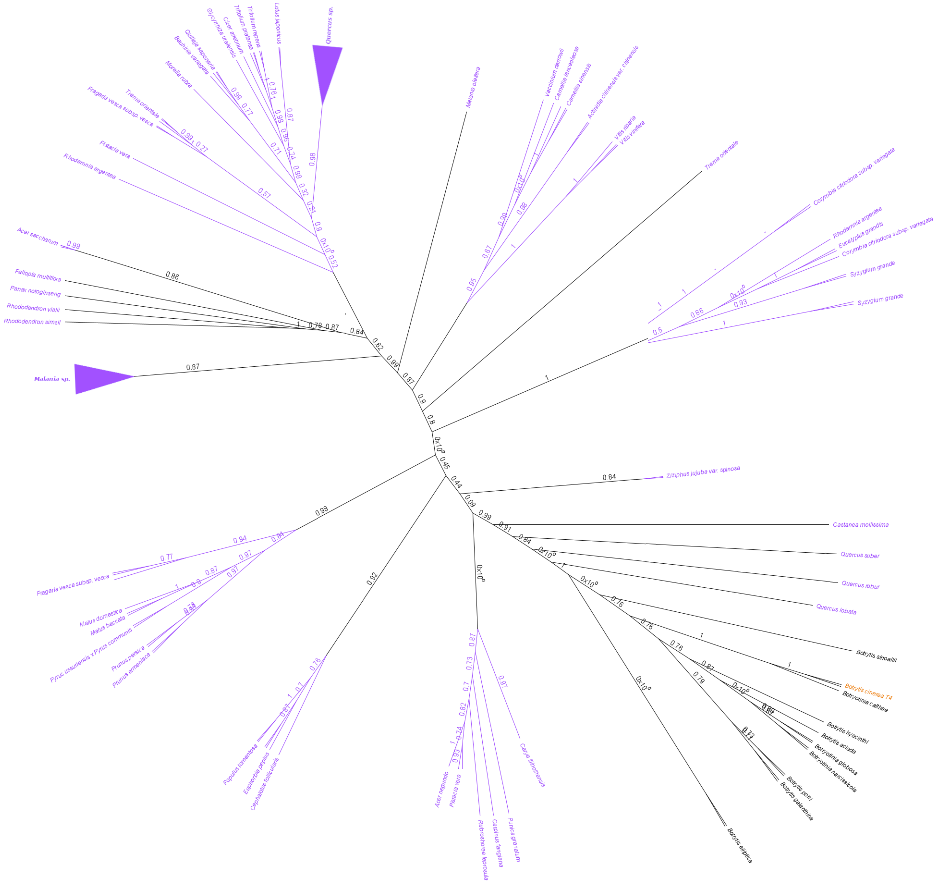

Figure S8. Phylogenetic tree illustrating the HGT 8 event. Fungal species are depicted in black, while non-fungal species are shown in purple, and the original species where the event was observed is indicated in dark yellow. Shimodaira-Hasegawa values are displayed next to the nodes, reflecting the support for the respective clades. For simplicity, some branches have been collapsed and are represented by triangles.

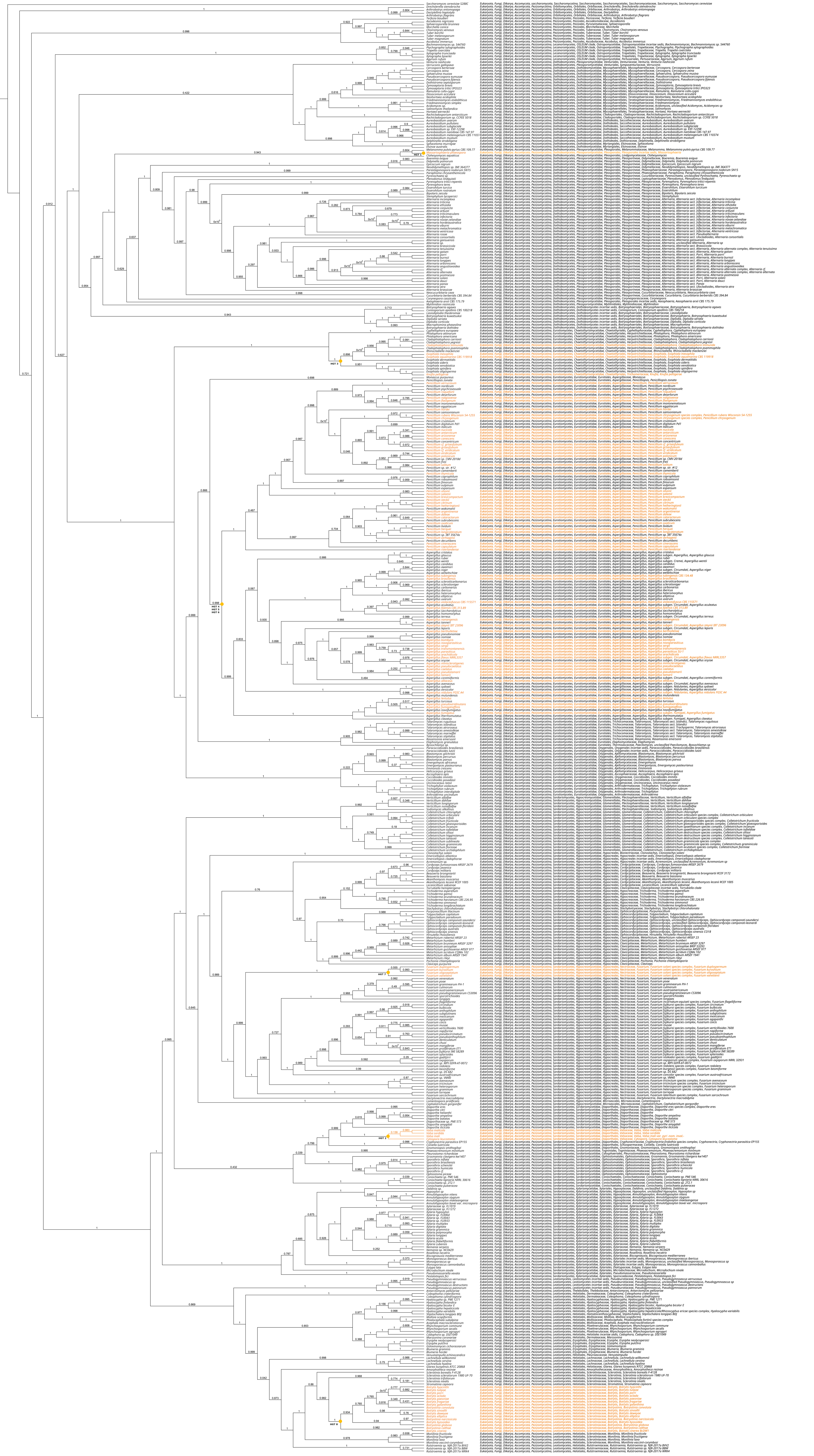

Figure S9. Species tree depicting horizontal gene transfer (HGT) events within Pezizomycotina. Yellow labels indicate taxa involved in HGT events. The minimum common ancestor for each HGT scenario is indicated by yellow circles. Eight HGT instances were identified: *Massariosphaeria phaeospora* (HGT 1), Valsaceae species (HGT 2), Chaetothyriales species (HGT 3), Aspergillaceae (HGT 4, 5 and 6), *Fusarium* genus (HGT 7) and *Botrytis* genus (HGT 8).

Shimodaira-Hasegawa values are displayed next to the nodes, reflecting the support for the respective clades.
